# Parallel Fish School Tracking Based on Multiple Appearance Feature Detection

**DOI:** 10.3390/s21103476

**Published:** 2021-05-17

**Authors:** Zhitao Wang, Chunlei Xia, Jangmyung Lee

**Affiliations:** 1 Department of Electronics Engineering, Pusan National University, Busan 46241, Korea; zhitao7379@pusan.ac.kr; 2 Yantai Institute of Coastal Zone Research, Chinese Academy of Sciences, Yantai 264003, China; clxia@yic.ac.cn

**Keywords:** zebrafish, SORT, Kalman filter, shape index, clustering

## Abstract

A parallel fish school tracking based on multiple-feature fish detection has been proposed in this paper to obtain accurate movement trajectories of a large number of zebrafish. Zebrafish are widely adapted in many fields as an excellent model organism. Due to the non-rigid body, similar appearance, rapid transition, and frequent occlusions, vision-based behavioral monitoring is still a challenge. A multiple appearance feature based fish detection scheme was developed by examining the fish head and center of the fish body based on shape index features. The proposed fish detection has the advantage of locating individual fishes from occlusions and estimating their motion states, which could ensure the stability of tracking multiple fishes. Moreover, a parallel tracking scheme was developed based on the SORT framework by fusing multiple features of individual fish and motion states. The proposed method was evaluated in seven video clips taken under different conditions. These videos contained various scales of fishes, different arena sizes, different frame rates, and various image resolutions. The maximal number of tracking targets reached 100 individuals. The correct tracking ratio was 98.60% to 99.86%, and the correct identification ratio ranged from 97.73% to 100%. The experimental results demonstrate that the proposed method is superior to advanced deep learning-based methods. Nevertheless, this method has real-time tracking ability, which can acquire online trajectory data without high-cost hardware configuration.

## 1. Introduction

Video-based animal collective behavior analysis, due to the high scientific values and a wide range of potential applications, become a hot research topic thanks to recent advances in the computer vision method. Zebrafish are widely adapted in many fields as an excellent model organism, such as in biology, neurology, and ecology research [1,2,3,4]. It is essential to obtain the accurate trajectory and rapid identification of each individual for quantitatively analyzing their collective behavior, thus, to discover new principles underlying these behaviors.

However, there are still many challenges, comparing to the pedestrian tracking, the most common application of multiple object tracking (MOT), such as the fish are indistinguishable to the human eye because of similar appearance, the appearance and shape may change tremendously while swimming, and the orientation free detection method is required because the top view commonly observed in the application of zebrafish tracking. To solve these difficulties, a series of computer vision tracking methods were developed. These works commonly consist of individual detection and movement tracking. 

In the detection stage, the previous methods generally fall into two categories: detecting based on blob and detecting based on appearance feature. The blob-based detecting methods extract moving regions as the candidates of targets by subtracting a background model. Subsequently, the methods involve extracting targets according to the pre-defined geometry characteristic from the candidates. For example, some techniques use a specific model of the animal body based on the head shape [5], the body geometry [6,7,8], or the symmetry axis [9]. These methods may miss the target while an occlusion event occurred and can only be applied for the targets geometrically compatible with the used model. The other approaches that detect targets rely on the appearance feature of the target. Comparing to the common MOT algorithms that focus on extracting highly valuable features and filtering out high response features in recent years [10], tracking zebrafish, all the targets with similar appearance, it is more important to achieve high accuracy detection during occlusion. Therefore, Qian et al. and Wang et al. proposed a novel fish head detection method based on scale-space Determinant of Hessian (DoH) [11,12], and Barreiros et al. proposed a detector based on a convolutional network to delimit the region of the fish heads to optimize individual fish detection [13]. In addition, Kaarthick et al. proposed a detector based on Histogram of Oriented Gradients (HOG) to detect the high-speed moving basketball players [14]. Since the deformation and illumination are still challenging for the method based on appearance features, Yue et al. proposed a tracking algorithm based on Resnet features and cascaded correlation filters to improve precision and accuracy [15]. In the above methods, tracking targets were represented as a single point or a blob that may miss the target during occlusion, thus increasing difficulty in the tracking stage.

In the second stage, tracking, the existing methods can be divided into two categories: tracking based on data association and tracking based on identification. The data association-based methods assign the detected target in the current frame to the corresponding tracker by minimizing the assignment cost to get the global optimized result. According to the information obtained in the detection stage, such as position, direction, and blob size, a cost function can be constructed; then, the association task becomes a global optimization problem [6,11,16,17]. The second category of tracking method is trying to correctly identify targets then perform the tracking of targets based on the identification. For example, Rodriguez et al. proposed identifying targets based on an intensity histogram and Hu moments [18]. In addition, convolutional neural network-based methods are proposed to identify targets with similar appearance [5,19,20,21]. However, these methods require high-resolution images, which are computationally intensive and may require access to future frame images; thus, they cannot be applied in real-time applications.

In addition, the occlusion among biological individuals is the greatest challenge of vision-based behavioral monitoring system. To address the problems caused by occlusion in the tracking stage, the strategy of most existing methods is assigning a detected target to a tracker stringently with the cost of generating more trajectory fragments, then performing a post process to link these trajectory fragments [19,20,21]. For offline applications, this strategy can improve the performance of the tracking system; however, it is not applicable in real-time monitoring. In addition, the false positive in the detection stage can be filtered out with some constraints or be ignored in the tracking stage. However, the false negative caused by occlusion in the detection stage is difficult to compensate and may decrease tracking accuracy.

To overcome the limitations of previous works, a parallel tracking scheme is proposed to enhance the tracking performance by reliable fish detection and individual tracking with multiple appearance features. The main contributions of this work are as follows:

1. A multiple feature detection method is developed to extract the fish head and center of the fish body. The proposed fish detector was robust in detecting and locating individual fishes from occlusions. The motion state and bending degree of the fish body could be obtained in the detection process.
2. The proposed parallel tracking scheme could estimate individual positions by examining the fish head and center of the fish body simultaneously. If the detector failed to locate the fish head or the fish body, the proposed tracker could follow the fish movement according to the other features. This was effective to improve the robustness of tracking, especially during occlusions.
3. Real-time tracking performance was achieved in the experiments. Since the proposed method required less computational cost, the maximal tracking efficiency reached 67.39 FPS.

## 2. Proposed Method

**Figure 1:**
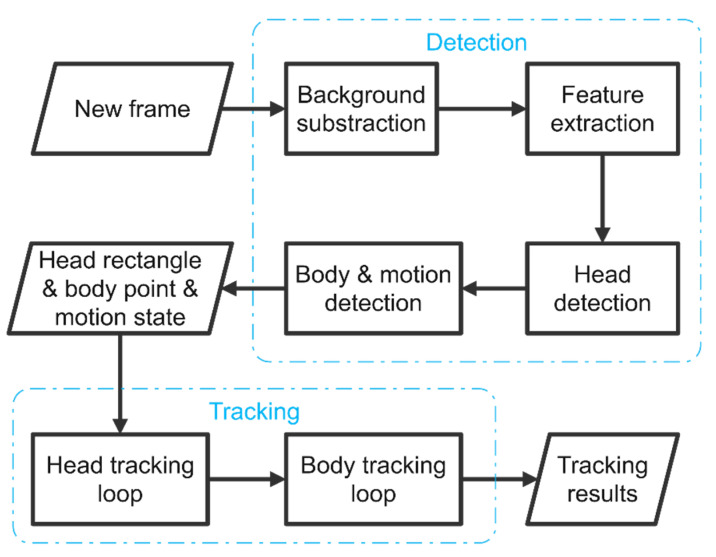
Flow chart of the proposed tracking scheme.

The proposed tracking scheme consists of two stages: individual fish detection and data association. The overall flowchart of the proposed tracking scheme is described in Figure 1. In the detection stage, fish images in the video frame are extracted by subtracting the background model. Subsequently, structural features of the fish image are extracted by shape index, which is derived from the eigenvalues of the Hessian matrix [22]. Taking these features into consideration, a multiple feature-based fish detection is developed to detect concave features, fish heads, and ridge feature on the fish body. The motion state of each individual is determined according to the ridge feature on the fish body. In the data association stage, a parallel tracking procedure is developed that tracks the fish head movement and fish body movement simultaneously according to the detected fish head, ridge position, and motion state information. The tracking process is implemented based on the framework of SORT [23] and optimized for multiple fish tracking tasks. The details of each step in the proposed tracking scheme are described in the following sections.

### 2.1. Fish Image Segmentation

**Figure 2:**
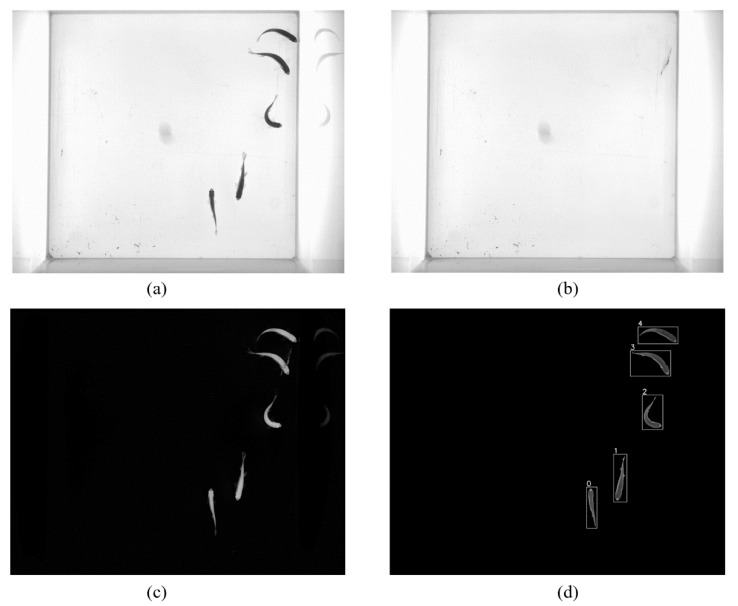
Fish segmentation process, (**a**) original image, (**b**) background image, (**c**) differential image, (**d**) blob detection and bounding boxes.

It is possible to segment moving regions from the background by subtracting a static background image because the laboratory environment is relatively stable, and the moving targets stay only for a short time in an area. As shown in Figure 2, firstly, the background image can be obtained using the time domain-based median filtering method [24] on the first n frames. Then, the moving regions can be segmented by setting a threshold for the differential image of the input image and the background image. In addition, a size constraint is utilized to remove most of the false positives. The detected moving region (blob) is written as $P_{t}^{i}=\left\{\left(x,y\right),w,h,P\right\}$, where t and i denote the frame index and blob index, (x,y) denotes the coordinate of the left top corner of the moving region bounding box, *w* and *h* denote the width and height of the bounding box, respectively, and P is a set of pixels of a moving region.

### 2.2. Structural Feature Analysis by Shape Index

The previous step obtains the image patches of moving region that may include multiple individuals during occlusion events. To detect each individual and estimate the motion state, the shape index algorithm [22] was employed to extract the local structural information of fish appearance. Considering the gray image as a 3D plane with intensities representing heights and using a Gaussian kernel with different standard deviation, the structural details in different scales can be represented by a single-valued measure of local curvature, which is derived from the eigenvalues of the Hessian matrix. The Hessian matrix is defined as:

(1) $$H\left(x,y\right)=\left[\begin{array}{cc} L_{xx} & L_{xy}\\ L_{xy} & L_{yy} \end{array}\right]$$

where $L_{xx}$, $L_{yy}$, and $L_{xy}$ are the convolution results of the Gaussian second-order derivatives at point (x,y). The eigenvalues of the Hessian matrix can be expressed as:

(2) $$\{\begin{array}{c} K_{1}=\frac{L_{xx}+L_{yy}+\sqrt{{\left(L_{xx}-L_{yy}\right)}^{2}+4L_{xy}{}^{2}}}{2}\\ K_{2}=\frac{L_{xx}+L_{yy}-\sqrt{{\left(L_{xx}-L_{yy}\right)}^{2}+4L_{xy}{}^{2}}}{2} \end{array}.$$

**Figure 3:**
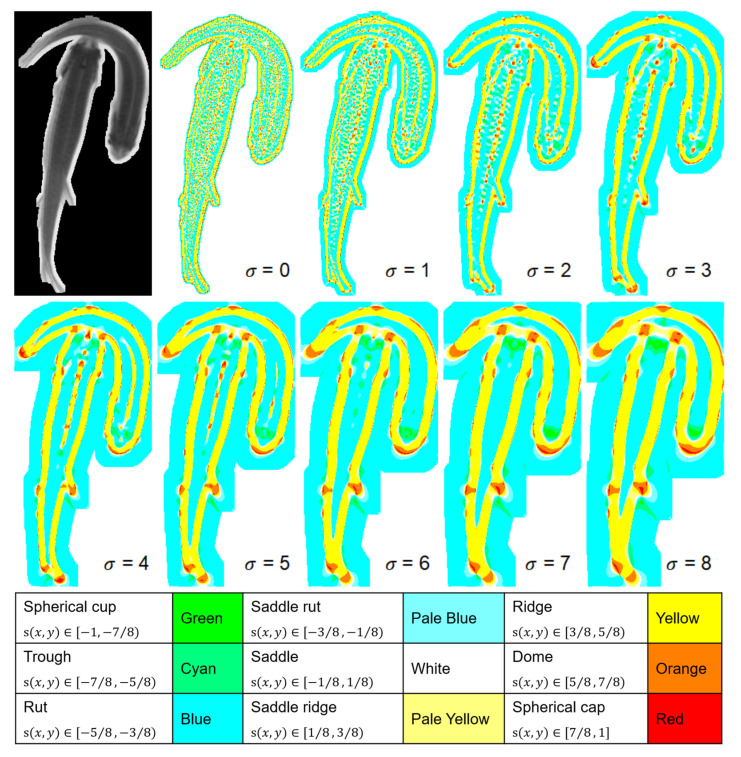
Shape index results image produced by Gaussian kernels with different σ values and the definition of shape index categories.

Finally, the shape index is defined as Equation (3) [22]:

(3) $$s\left(x,y\right)=\frac{2}{\pi }arctan\frac{K_{2}+K_{1}}{K_{2}-K_{1}}\left(K_{1}\geq K_{2}\right)$$

where the shape index value s(x,y) at point (x,y) is mapped on the segment [−1,1]. The shape index is characterized into 9 categories according to the geometry appearance [22]. The names and value ranges of each category are given in Figure 3. For example, when s(x,y) valued in [−1,−7/8), the pixels show a spherical cup shape in the shape index image, and the shape index image is colored according to the shape index category of each pixel.

According to the shape index, all the points in the blobs are classified into 9 categories. Examples of shape index processing with different Gaussian kernels are illustrated in Figure 3.

Different structural features can be extracted by using different scales of σ values. For example, the ridge of the fish body could be obtained when σ = 4, where the pixels of the center part of fish are brighter than the surrounding pixels. If σ = 8 is given, the fish head area presents a concave region that contains smaller values than the neighboring pixels.

### 2.3. Multiple Feature Extraction

After extracting the shape index in different scale spaces, the local structural information can be represented as a single value at each pixel. In the case of tracking zebrafish, the cup and ridge feature are used to detect target individuals, redundantly, and the motion state of fish is represented as a single value according to the curvature of the ridge line on the fish body.

**Figure 4:**
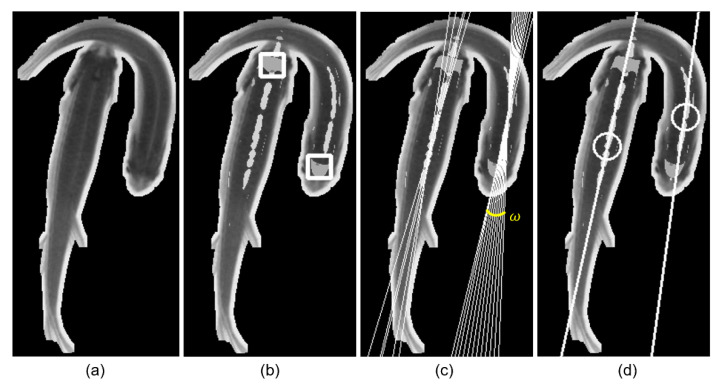
(**a**) Blob image, (**b**) head, (**c**) motion state, (**d**) body point.

Firstly, as shown in Figure 4b, a bounding box of the concave area is used to represent the head region of the fish, and the false positives are filtered out with a size threshold. The bounding box is denoted as $B=\left[\left(x_{0},y_{0}\right),\left(x_{1},y_{1}\right)\right]$, where $\left(x_{0},y_{0}\right)$ and $\left(x_{1},y_{1}\right)$, are the coordinates of the left top corner and right bottom corner of the bounding box, respectively.

Secondly, as shown in Figure 4c, the ridge feature is represented by a bunch of straight lines, which are detected by the Hough Transform. Each one of the lines can be represented as (ρ,θ), where ρ is the perpendicular distance from the origin to the line, and θ is the angle formed by this perpendicular line and the horizontal axis measured counterclockwise. Then, since there may be more than one individual in a detected blob during the occlusion event, all the lines are grouped into clusters according to (ρ,θ) using the Density-Based Spatial Clustering of Applications with Noise (DBSCAN) algorithm [25], which views clusters as areas of high density separated by areas of low density. The clustering result is shown in Figure 4d; the straight line indicates the average line of the lines in a same cluster. The coordinate of the circle center represents the position of the fish body, which is the intersection point of the lines within the same cluster.

After extracting and analyzing the local structural feature, numerically, for the detected features, we define the detection of fish head $h_{t}^{i}$ and fish body $b_{t}^{i}$ as follows:

(4) $$h_{t}^{i}=B$$

(5) $$b_{t}^{i}=R,\omega$$

where t and i are the frame index and blob index, respectively; B is the bounding box representing the fish head; R is the coordinate of the intersection of ridge lines representing the fish body position; ω is the angle between the ridge line with a maximum angle and minimum angle and represents the motion state. The smaller the ω is, the steadier the motion is; on the contrary, the bigger the ω is, the more unstable the motion is.

### 2.4. Parallel Tracking Using Multiple Features

**Figure 5:**
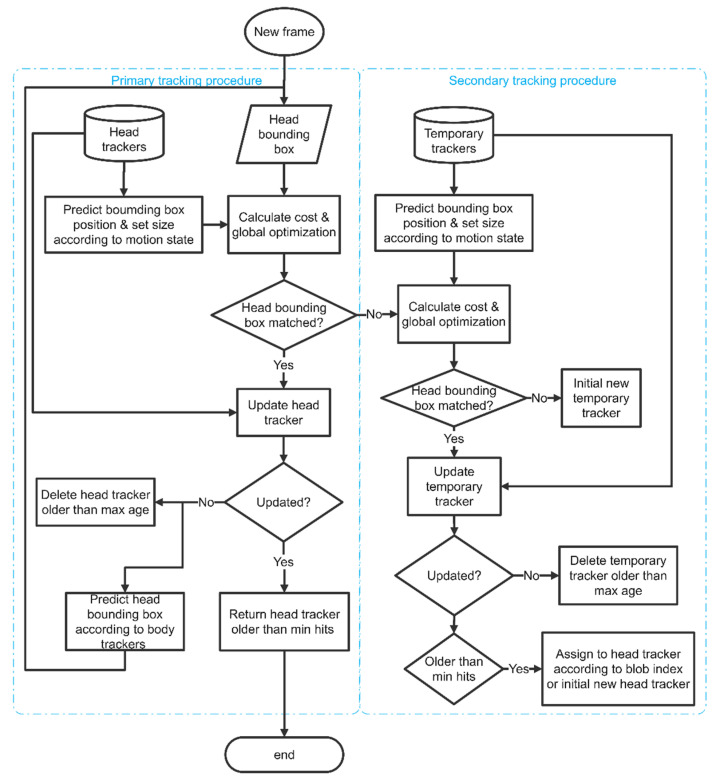
Flow chart of head tracking loop.

Since the occlusion event occurs frequently and randomly when a fish swims, the incomplete appearance of a fish can result in a missed detection or false positives, which decrease tracking accuracy and stability. Multiple features of a fish, i.e., fish head, ridge point on body, and motion state, are obtained in the previous steps. In this work, a parallel tracking scheme is developed by combining the multiple fish appearance features and motion features. The parallel tracking is implemented by tracking the fish head and fish body simultaneously. The head tracker and body tracker run parallelly to record the individual trajectories. In this work, the fish head is considered as the primary feature of the fish, and the tracker follows the fish head in preference to the fish body. Since the fish head presents a rigid shape without scale and shape variations, the structure of the fish head tracker is illustrated in Figure 5. The tracking algorithm is inspired by the SORT framework and optimized for a multiple animal tracking task. The basic idea of the proposed tracking scheme is assigning detected targets to existing trackers according to the assignment cost matrix; each tracker’s bounding box geometry is estimated by predicting its new location in the current frame via a Kalman filter framework [26]. At the beginning of processing each frame, head trackers predict positions by Kalman filter and bounding box size for each individual according to their motion state. Subsequently, the intersection-over-union (IOU) between the detected heads and predicted bounding box of each individual is calculated [23] and a cost matrix of IOU is constructed as shown in Equation (6).

(6) $$C=\left[\begin{array}{ccc} c_{11} & \cdots & c_{1m}\\ \vdots & \ddots & \vdots \\ c_{n1} & \cdots & c_{nm} \end{array}\right]$$

where *c_nm_* denotes the IOU distance between the detected target m and predicted bounding box of tracker *n* in the current frame. *n* and *m* are the number of trackers in the previous frame and the number of detected targets in current frame, respectively. The Hungarian algorithm is employed to solve the optimal assignment problem [27]. The objective function is:

(7) $$\delta =\underset{A}{\min}\sum^{\;}\sum^{\;}c_{nm}*A_{nm}$$

subject to:

(8) $$\underset{n}{\sum }A_{nm}=1\;and\;\underset{m}{\sum }A_{nm}=1$$

By the optimal assignment, each detected head should be assigned with an ID, and each tracker should be associated to a detected head. However, unassigned heads and unassociated trackers may occur when false positives and misdetection happened in the detection process. The proposed tracking scheme should further deal with these failures. A temporary tracking procedure is employed to examine the unassigned detections (Figure 5). The temporary trackers predict positions and assigns IDs to the detections in the same manner as head trackers. If a detection still could not be assigned with an ID, a new temporary tracker will be initiated from this detected head. If a temporary tracker could not be associated to any detections for a time period longer than the max age, it should be removed as a noise. If a temporary tracker succeeds in associating to detections more than the minimal hits, it should be considered as a head tracker. This head tracker should be linked to a lost ID when there are one or more IDs lost in the tracking procedure.

**Figure 6:**
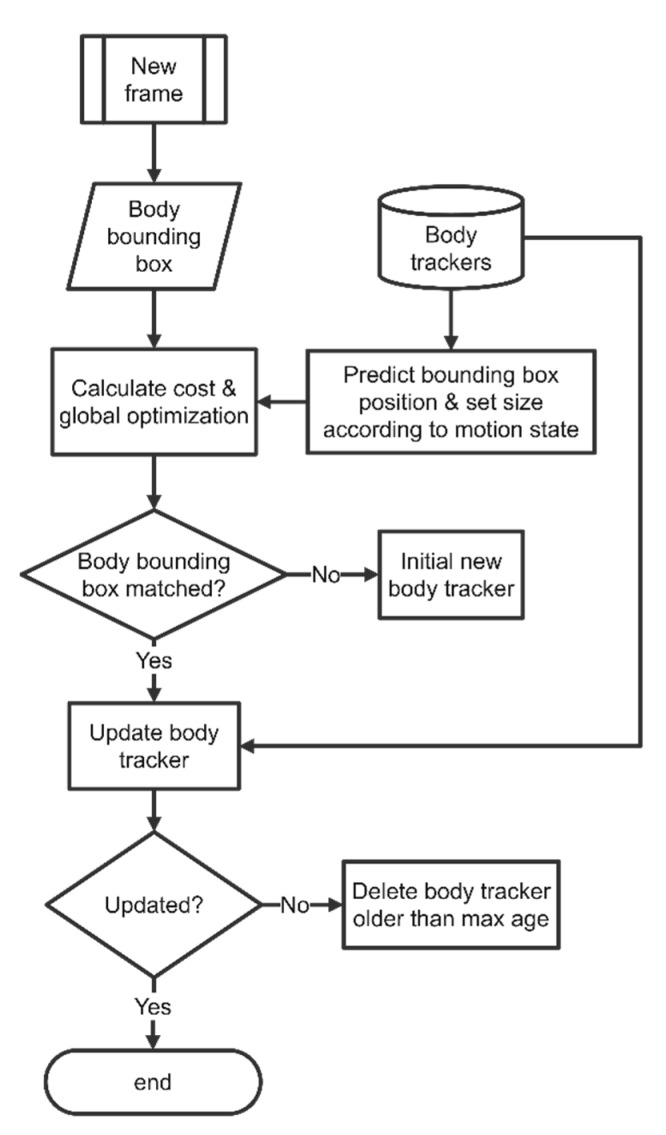
Flow chart of body tracking loop.

For the unassociated head trackers, the body tracking procedure will be employed to find the target individuals. When the head tracker loses tracking targets more than the max age, the tracker should be terminated and removed. The body tracking procedure is described in Figure 6. It has the same prediction and association process on body rectangles, which are obtained in the detection results. The head tracker and body tracker record the head position and center point of the fish body concurrently. Once the head tracker fails to locate the fish head, the body tracker could provide the center point of the fish body to estimate the fish head positions and update the head trackers.

### 2.5. Evaluation Metrics

In this work, individual fish detection and tracking performance of multiple fishes are evaluated separately. Detecting individual fishes is the basic requirement for implementing multiple individual tracking. Individual detection accuracy determines the tracking performance. For example, detecting occluded fishes could efficiently improve the tracking ability of a group of fish. The precision-recall analysis, which has been widely adopted to the evaluation of object recognition and detection algorithms, is applied to assess individual detection accuracy by the proposed method. The calculation of precision and recall is given in Equations (9) and (10).

(9) $$Precision=\frac{true\;positive}{true\;positive+false\;positive}$$

(10) $$Recall=\frac{true\;positive}{true\;positive+false\;negative}$$

where true positive is the total number of correctly detected targets in all frames. False positive is the total number of incorrectly detected targets. Usually, false positives are from the mirrored image on the acrylic tank wall or occlusion. False negative is the total number of missed targets. Therefore, occlusion probability was used to measure the probability that one individual is occluded in a frame image; the calculation is given in Equation (11):

(11) $$Occlusion\;rate=\frac{number\;of\;occlusions}{number\;of\;individuals\;*\;number\;of\;frames}.$$

In addition, to qualify the performance of the proposed detection method during an occlusion event, the occlusion detection ratio was utilized. The calculation of occlusion detection is given in Equation (12):

(12) $$Detection\;rate\;from\;occlusions=\;\frac{correct\;number\;of\;occlusion\;detection}{number\;of\;occlusions}.$$

The tracking performance of the proposed method is evaluated similarly to the evaluation of detection performance. The correct tracking ratio (CTR) and correct identification ratio (CIR) were used. The CTR as shown in Equation (13) describes the percentage of frames correctly tracked for each fish. The CIR as shown in Equation (14) describes the percentage of correct identification of all fish after an occlusion event. Occlusion is one of the greatest concerns in multiple objects tracking, which is the significant disturbance in decreasing the tracking accuracy. The CIR represents the stability of the tracking scheme against occlusions, and the high CIR value indicates that the tracking scheme is robust to occlusion. In addition, the total computational time and the frequency of ID switch after occlusion were measured.

(13) $$CTR=\frac{\sum^{\;}\left(number\;of\;correct\;frames\;of\;a\;single\;target\right)}{number\;of\;individuals\;*\;number\;of\;frames}$$

(14) $$CIR=\frac{\;Times\;That\;ALll\;Fish\;Get\;Correct\;Identity\;After\;occlusion}{number\;of\;occlusions}$$

## 3. Experiments and Results

### 3.1. Experimental Conditions

**Figure 7:**
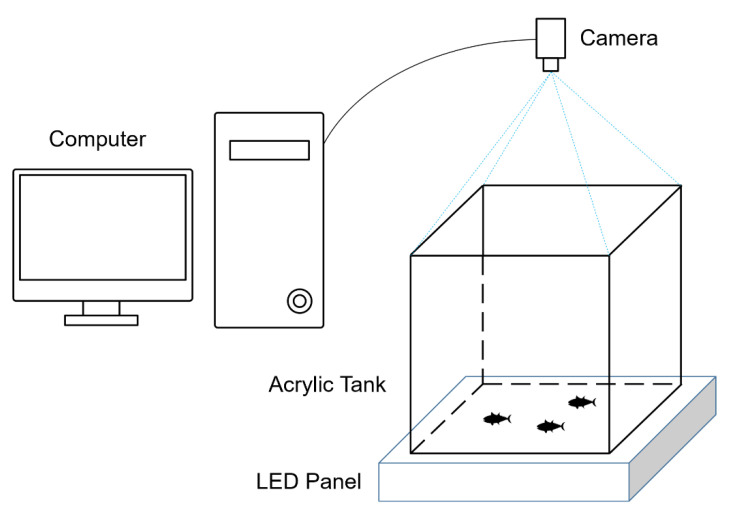
Observation system setup.

Fish observation tests were conducted in our customized behavioral observation facility to collect video data of zebra fish. The fish observation facility was designed to observe the two-dimensional movement of fish as illustrated in Figure 7. A high-resolution digital camera was placed over the observation arena and the height of camera was adjusted to cover the entire area of the arena. The digital camera was produced by Hikvision (MV-CA050-20UM) and the image resolution was 2592 × 2048 pixels. The observation arena was made by a square acrylic tank of size 20 cm × 20 cm. A white LED panel provided backlighting illumination from the bottom of the acrylic tank to remove the reflection from water surface. Adult zebra fishes were chosen in the tests. The zebra fishes were approximate 3–4 cm long and water depth was set to 10cm.In the experiment, video clips of 5 zebra fish were recorded and two of them were chosen as the test data (D1 and D2 in Table 1). These video data could be downloaded in the Supplementary Materials.

The proposed tracking scheme was implemented by Python and image processing libraries such as OpenCV and Skimage. All the tests were conducted on a personal computer configured with an AMD CPU 3800X@3.9 GHz, 16G RAM under Ubuntu 20.04.

### 3.2. Experimental Data

In this work, seven video clips from different sources were collected to evaluate the reliability and robustness of the proposed tracking scheme. Details of these video clips are presented in Table 1. D1 and D2 were taken with our observation facility as described in Section 3.1. D3 and D4 were the experimental video in [6], while D5–D7 were chosen from [20]. In the datasets, the frame rate was from 32 to 100 fps, the number of target individuals was from 5 to 100, and individual size ranged from 240 to 9800 pixels. In addition, the number of occlusion events was examined to investigate the influence of occlusion on tracking performance. Every occlusion event of fishes was counted in all the frames in each test data. Occlusion frequency is one of the major concerns in tracking a group of fishes. Occlusion frequency is mainly determined by fish density in the observation arena and movement patterns. For example, a small number of fish live in a relatively large tank that shows a low probability of occlusion. On the other side, if the fishes are showing some special behavioral pattern, such as chasing and biting, it may increase the occlusion frequency [28]. In our datasets, the most occlusions occurred in D4, since it had 20 medium-sized individuals in a relatively small observation arena. Although 100 individuals observed in D7, the individual fish was rather small, the occlusion frequency was not the most.. Moreover, the fishes in D7 presented group behavior that all fishes were swimming along the same direction in a certain speed.

### 3.3. Evaluation of Individual Detection

The accurate detection of fish is a key step for tracking individual movement. The accuracy of the proposed multiple-feature fish detection was evaluated by precision–recall analysis (Table 1). Precision of individual detection was in the range of 98.62–100.00% and recall value was 99.57–99.99%. The average precision of all datasets was 99.7%, indicating that a small proportion of false positives were incorrectly identified as fishes. Meanwhile, the average recall was 99.81%, which means that only 0.19% of the individuals were missed. One of the advantages of the proposed fish detection method was the ability to identify individuals from occlusions. Therefore, the accuracy of detecting individuals from occlusions was evaluated. The detection rates from occlusions were between 93.42% and 99.28%. It is notable that the average detection rate of occluded individuals was 97.5%, which proves that the proposed fish detection method is reliable when occlusion occurs. On the other side, Table 2 shows that the precision value was decreased with an increasing occlusion rate because false positives were produced when occlusions occurred; the tracker needs to be able to filter the false positive detections.

Moreover, the computational costs were assessed by examining the time consumption of the fish tracking scheme (Table 2 and Table 3). The computational costs of individual detection are presented in Table 2. The computational time of every frame was measured in each of the videos, which varied from 24.83 to 173.45 ms per frame. Accordingly, the frame rates of individual detection were obtained as 5.77–41.02 frames per second (FPS). The average frame rate in the whole dataset was 25.50 FPS. The detection time was dependent on the number of individuals, frequency of occlusions, and individual size. Dataset D7 consumed the longest computational time (173.45 ms) since 100 individuals needed to be detected. D4 cost a similar time (170.80 ms) to detect only 20 individuals because the most occlusions appeared and the fish size was approximately 10 time larger than that of D7. The processing of D5 was the fastest (12.68 ms) and reached to 78.86 FPS because of the small fish size and a smaller number of individuals.

In addition, the recall ratio that plays a more important role in the application of group animal tracking can limit the overall performance of the tracking system. Normally, the recall ratio will decrease when occlusion probability increases, especially for the methods that highly rely on appearance features. Therefore, it is necessary to increase the detection accuracy during an occlusion event.

### 3.4. Performance of the Proposed Tracking Scheme

Tracking performance of the proposed scheme was further evaluated by analyzing CTR, CIR, and ID switch (IDS) of the tracking results. As presented in Table 3, the CTRs in each of the test videos exceeded 99.06%, and the average value of CTR was 99.25%. CTR represents the overall tracking accuracy, while CIR investigates the tracking stability against occlusions. The CIR value ranged from 97.73 to 100.0%, and the average CIR was 99.45%. The proposed tracking scheme showed outstanding tracking accuracy when occlusion occurred. The movement trajectories were all correctly assigned to every individual in D1–D4. Especially, D4 presented the highest frequency of occlusions. Moreover, ID switch was also effectively eliminated, since most ID switch were caused by occlusions. In D5–D7, ID switch appeared when CIR decreased. The experimental results showed that CIR had less impact on CTR, particularly in D1–D4 where CIR was 100.0%. CTR measures the proportion of individuals with a correctly assigned ID. The error of CTR was from the incorrect assignments generated by detection failure. As discussed in Table 2, misdetection was evaluated by recall values. In addition, the severely occluded fishes were also counted as incorrect assignments when calculating the CTR. Since only incomplete appearance information could be observed from the severely occluded fishes, they were not considered as the detection targets. Therefore, CTR was slightly lower than the recall values of detection despite CIR reaching 100.00%. It explains that CTR decreased when the occlusion rate increased (Table 3). The processing speed of the proposed tracking scheme was further analyzed in Table 3. The running time of data association, which assigns an ID to each individual, was measured. The data association time varied from 0.75 to 10.65 ms, and the results showed that the association time was determined by the number of tracking targets. The overall time consumption of the tracking process included the detection time and data association time. The overall tracking costed 13.76 to 184.10 ms per frame among all the datasets. The fastest processing was 67.39 FPS observed on D5, and the most time consumption was 5.13 FPS on D7, since the tracking targets reached 100 individuals, which required a longer time for individual detection and ID assignment.

To prove the advanced ability of the proposed fish tracking scheme, a comparison test was carried out with a state-of-the-art fish tracking method, IDTracker.ai [20]. All the datasets from three different sources were chosen in the comparison tests. The tracking accuracy of the proposed tracking scheme and IDTracker.ai are presented in Table 4. The best tracking accuracy was marked in bold font. In D1 and D2, our method showed 2.15% and 1.77% higher values in averaged CTR and CIR than IDTracker.ai, respectively. In D3 and D4, the proposed method presented much higher CTR (99.17% and 98.60% for D3 and D4) values compared with IDTracker.ai (93.37% and 88.63%). Since the texture feature was not clearly captured in D3 and D4, the tracking accuracy was significantly decreased in IDTracker.ai. In D5 and D6, both methods showed CTR > 99.0%, while the proposed method was 0.52% lower than IDTracker.ai on averaged CTR. Comparing with IDTracker.ai, the proposed method presented 0.51% lower CIR in D5 and 0.36% higher CIR in D6. The result of D7, the proposed method, and IDTrakcer.ai presented similar performance. The proposed method was 0.91% higher than IDTracker.ai in CTR and 0.36% higher in CIR. On the overall tracking performance, the proposed method outperformed IDTracker.ai in both of CTR and CIR. The average CTR and CIR of the proposed method were 99.25% and 99.45%, which showed 2.74% and 0.86% higher values than that of IDTracker.ai. It is worth noting that the proposed method could produce a robust tracking performance on compressed videos or low-quality videos. In the tests, compressed videos could not reduce the tracking accuracy of the proposed method. However, IDTracker.ai requires uncompressed video (raw RGBA format) to maintain the tracking accuracy. The tracking by IDTracker.ai might fail in compressed video, especially when dealing with 100 individuals in D7.

## 4. Discussion

The randomness and frequent occlusion is still challenging in multiple animal tracking application. The identification of individual fishes is important and effective to improve the performance of tracing multiple individuals. Previous works reported that loose tracking targets could increase the probability of ID switch, and a trajectory re-linking process is necessary to match the trajectories before and after occlusions. In some cases, the trajectory re-link could not accurately match the tracklets and switch the trajectories. In this work, detecting occluded individuals is proposed to improve the tracking stability against occlusions. Detection based on fish head and body could ensure the robustness of detection. The detection and tracking can work properly even if a part of the fish is unseen. Outstanding detection accuracy is achieved in that the average precision was 99.70%. Only 0.30% false positives were incorrectly identified. These false positives could be eliminated in the tracking procedure, since usually, false positives could not last for a long time. The tracking procedure examines each tracker and removes the trackers that cannot continuously be associated to any detections.

The parallel tracking is another contribution of this work that improved the robustness of tracking multiple fishes with similar appearance. Multiple feature fish trackers, i.e., head tracker and body tracker, work independently to follow the fish movement. The head tracker is considered as the primary tracker, and the body tracker is the secondary tracker. The body tracking procedure can provide additional association information when the primary tracker fails to locate the tracking targets—for example, when the fish head is severely occluded during an occlusion event. In addition to the head-tracking procedure, a temporary tracking procedure is employed for the unassigned detections. The temporary tracking procedure focuses on cautiously initializing a new tracker and ensuring the reliability against the influence of a false positive. The temporary tracking procedure guarantees that the detected targets are matched with more reliable trackers, preferentially.

The advantages of our method are that it is accurate, fast, and computational inexpensive. The proposed tracking scheme outperformed the state-of-the-art tracking method, IDTracker.ai., which is an offline tracking scheme. Offline techniques usually achieve a significantly higher accuracy than online methods because future frames of the video clip were involved in the tracking algorithm. Many offline methods require long time for analyzing video. For example, IDTracker.ai took hours for analyzing the datasets in Table 4. The proposed tracking scheme is an online tracking method and could be applied to real-time behavioral tracking. Although future frames are not available for the online tracking scheme, the proposed tracking scheme presented impressive tracking accuracy due to the advantages of detecting occluded fish and the parallel tracking scheme.

In the experiments, we found that fish can change their body shape in turning behavior. The fish body could bend at a large angle and return to the straight shape in a very short time. The sudden change of fish shape might cause blurring images and brought difficulties to the trackers. Therefore, a short exposure time, e.g., 2 ms, is recommended to ensure the image clarity. Moreover, Kalman filter may fail to predict the motion state of a fish in rapid transition. This issue was solved by using a bounding box with variable size estimated according to the motion state. The experimental results showed that the tracking accuracy could be decreased with low frame rates. It should be noticed that when the frame rate is over 60 FPS, the CIR ratio reaches up to 100%. A high frame rate indicates high sampling frequency; therefore, fish movements turn to linear and Kalman filter can predict the individual movement more accurately. Consequently, a higher IOU between predictions and observations could be obtained, which can promote the accuracy of assigning IDs to each target. When the frame rate is low, the association procedure may result in more tracklets and a higher possibility of incorrect identification.

In future work, the proposed method should be applied to real-time monitoring and evaluate the tracking performance for long-term monitoring. Optimization of the proposed tracking scheme should be conducted to further reduce the computational time, for example, by introducing parallel computing in the calculation or developing GPU-based version.

## 5. Conclusions

This paper proposes a computational effective and accurate scheme to track a group of zebrafish. A novel multiple appearance feature detection method that requires no information of the shape of the animal has been proposed to increase the detection accuracy when an occlusion event occurs, and a single valued motion state is proposed to reduce the tracking error caused by prediction failure of Kalman filter. Moreover, the SORT algorithm has been modified to meet the requirements of zebrafish tracking. The improved tracking method consists of two parallel tracking loops: one for the first feature tracking and the other for the second feature. In the detection stage, two independent features are utilized: one represents the fish head region and the other represents the fish body. Based upon the features of the fish body and head, the intensity of motion of the fish has been approximated as a single-valued motion state. According to the motion state, the size of the detected bounding box is also adjusted. By the proposed scheme, the probability of misdetections has been reduced a lot for the occlusion cases, which increases the tracking accuracy. When the motion of the fish is rapid, the possibility of Kalman prediction failure is increased. To overcome this problem, the size of the bounding box is increased. The experiments have been performed using seven datasets of three sources with different fish sizes, different collective sizes, up to 100, and different frame rate/resolutions. The superior performance of up to 99.45% of CIR and 99.25% of CTR on average have been achieved by the proposed scheme. The results demonstrate that the proposed tracking system is robust and accurate.

## Figures and Tables

**Table 1:** Description of experimental datasets.

Dataset	Total Length (Frame)	Frame Rate per Second (FPS)	Image Resolution (Pixel)	Number of Fish	Individual Size (Pixel)	Frequency of Occlusions
D1	240	60	2592 × 2048	5	6500–9800	95
D2	300	60	2592 × 2048	5	7000–9800	279
D3	300	100	2040 × 2048	10	4000–6000	198
D4	200	100	2040 × 2080	20	2700–6400	546
D5	500	32	1920 × 1080	8	320–480	198
D6	500	32	3712 × 3712	10	450–700	277
D7	200	32	3584 × 3500	100	240–560	220

**Table 2:** Performance analysis of the proposed fish detection method.

Dataset	Precision (%)	Recall (%)	Occlusion Rates (%)	Detection Rate from Occlusions (%)	Computational Time per Frame (ms)	Frame Rate of Detection (FPS)
D1	100.00	99.75	7.92	97.89	58.83	17.00
D2	98.62	99.87	18.60	99.28	67.43	14.83
D3	99.80	99.57	6.60	93.43	65.87	15.18
D4	99.78	99.78	13.65	98.35	170.80	5.85
D5	99.73	99.85	4.95	96.97	12.68	78.86
D6	99.98	99.86	5.54	97.47	24.38	41.02
D7	99.99	99.99	1.10	99.09	173.45	5.77
Average	99.70	99.81	8.34	97.50	58.83	25.50

**Table 3:** Tracking performance analysis of the proposed tracking scheme.

Dataset	CTR (%)	CIR (%)	IDS	Occlusion Rates (%)	Data Association Time per Frame (ms)	Overall Tracking Time per Frame (ms)	Frame Rate of Tracking (FPS)
D1	99.42	100.00	0	7.92	0.75	59.58	16.58
D2	99.33	100.00	0	18.60	0.67	68.10	14.54
D3	99.17	100.00	0	6.60	1.27	67.14	14.62
D4	98.60	100.00	0	13.65	2.25	173.05	5.70
D5	99.33	99.49	1	4.95	1.08	13.76	67.39
D6	99.06	98.92	3	5.54	1.28	25.66	37.12
D7	99.86	97.73	5	1.10	10.65	184.10	5.13
Average	99.25	99.45	/	8.34	2.56	84.48	23.01

**Table 4:** Comparison results with IDTracker.ai (The best results are presented in bold font).

Datasets	CTR (%)		CIR (%)	
	Proposed	IDTracker.ai	Proposed	IDTracker.ai
D1	**99.42**	96.92	**100.00**	97.89
D2	**99.33**	97.53	**100.00**	98.57
D3	**99.17**	93.37	**100.00**	98.99
D4	**98.60**	88.63	**100.00**	99.27
D5	99.33	**99.68**	99.49	**100.00**
D6	99.06	**99.74**	**98.92**	98.56
D7	**99.86**	99.69	**97.73**	96.82
Average	**99.25**	96.51	**99.45**	98.59

## Data Availability

Not applicable.

## Supplementary Materials

The following are available online at https://www.mdpi.com/article/10.3390/s21103476/s1. The fish observation video data produced in this work (D1 and D2) is available for public access at: https://drive.google.com/drive/folders/1EdfAsCJGuFAnEmoW2Be4ZEyButTNvDyjN?usp=sharing (accessed on 17 May 2021).
